# TOP2A^high^ is the phenotype of recurrence and metastasis whereas TOP2A^neg^ cells represent cancer stem cells in prostate cancer

**DOI:** 10.18632/oncotarget.2411

**Published:** 2014-09-08

**Authors:** Xuefeng Li, Yunying Liu, Wenqi Chen, Yuxiang Fang, Huiming Xu, Helen He Zhu, Mingliang Chu, Wang Li, Guanglei Zhuang, Wei-Qiang Gao

**Affiliations:** ^1^ State Key Laboratory of Oncogenes and Related Genes, Renji-MedX Stem Cell Research Center, Ren Ji Hospital, School of Medicine, Shanghai Jiao Tong University, Shanghai 200127, China; ^2^ School of Biomedical Engineering & Med-X Research Institute, Shanghai Jiao Tong University, Shanghai 200030, China; ^3^ Department of Neurology, The Sixth People's Hospital Affiliated to Shanghai Jiaotong University, Shanghai 200240, China

**Keywords:** prostate cancer, cancer stem cells, TOP2A, recurrence, metastasis

## Abstract

Recurrence and metastasis are the main causes of death for prostate cancer patients and cancer stem cells (CSCs) are proposed to play important roles in cancer recurrence and metastasis. It is generally thought that genes upregulated in recurrent/metastatic disease are likely biomarkers of CSCs. Hence we analyzed multiple microarray datasets on prostate tumor tissues to identify upregulated genes associated with cancer recurrence/metastasis, and tried to explore whether those genes were true biomarkers of prostate CSCs. Our results indicated that TOP2A was the most highly upregulated gene in recurrent/metastatic prostate cancer, and its high expression was positively correlated with poor prognosis in patients. Using a promoter reporter system, we unexpectedly discovered enrichment of CSCs in TOP2A^neg^ cells. Compared to TOP2A^high^ cells, TOP2A^neg^ cells formed spheres and tumors more efficiently, and became enriched in the presence of stresses. Analysis of cell divisions by time lapse imaging indicated that more slow-cycling cells were observed in TOP2A^neg^ cells while the proportion of abnormal divisions was higher in TOP2A^high^ cells. Our studies demonstrate that TOP2A^high^ is the phenotype of recurrence/metastasis but TOP2A^neg^ cells show slow cycling and have CSCs properties in prostate cancer, which has significant implications for prostate cancer therapy.

## INTRODUCTION

Prostate cancer is one of the most common cancers worldwide. According to recent global cancer statistics, it is the second most diagnosed and sixth leading cause of cancer deaths in males [1]. Although early stage prostate cancer can be surgically excised and effectively treated by androgen blockade, chemotherapy or radiotherapy, recurrent and metastatic diseases are common and deadly. Over the past decades, a large number of studies have focused on recurrent and metastatic prostate cancer (referred to as secondary prostate cancer hereafter), which is often androgen-independent and chemotherapy-resistant. Several mechanisms that may lead to tumor recurrence/metastasis have been proposed, including the amplification or mutation of androgen receptor [2–4], expression of multidrug resistance gene [5, 6], epithelial-mesenchymal transition (EMT) [7–9] and cancer stem cells (CSCs) or cancer stem cell-like cells [8, 10–12].

CSC model was originally introduced by Mackillop et al. [13] and validated in acute myeloid leukemia (AML) for the first time in 1997 [14]. In this model, cancers are supposed to retain hierarchical organization in much the same way as normal tissues and CSCs constitute a small subset of tumor cells, which are functionally distinct from non-CSCs by their ability to seed new tumors. CSCs have been subsequently identified in a variety of human cancers, such as breast cancer [15], brain cancer [16], pancreatic cancer [17], liver cancer [18], and prostate cancer [19]. Therefore, identification of novel markers for CSCs is of importance and may offer more effective therapies for cancer patients.

In this study, we systematically analyzed genes upregulated in secondary prostate cancer and identified TOP2A to be at the very top of the list. TOP2A encodes topoisomerase IIa (topoIIa), an enzyme involved in DNA replication, transcription, recombination, and chromatin remodeling [20]. It plays an important role in DNA synthesis and transcription and has been implicated in a variety of human cancers [21]. It is usually assumed that CSCs are enriched in relapsed or disseminated tumors, and genes upregulated in recurrence/metastasis are likely markers for the CSCs [22–24]. Therefore, we further investigated whether TOP2A was a potential CSC marker in prostate cancer. Surprisingly, although TOP2A^high^ (high expression of TOP2A) cells were highly proliferative and were associated with recurrence/metastasis in prostate cancer, CSCs were enriched in a small minority which was TOP2A^neg^ (undetectable expression of TOP2A by FACS in promoter reporter system). These cells displayed slow-cycling, higher tumorigenic potential and were more resistant to chemotherapy and other stresses. Therefore, our findings argue for novel therapies targeting TOP2A^neg^ cells, in combination with conventional de-bulking strategies, to eradicate all tumor cells in prostate cancer patients.

## RESULTS

### Upregulation of TOP2A expression in secondary prostate cancer

**Figure 1:**
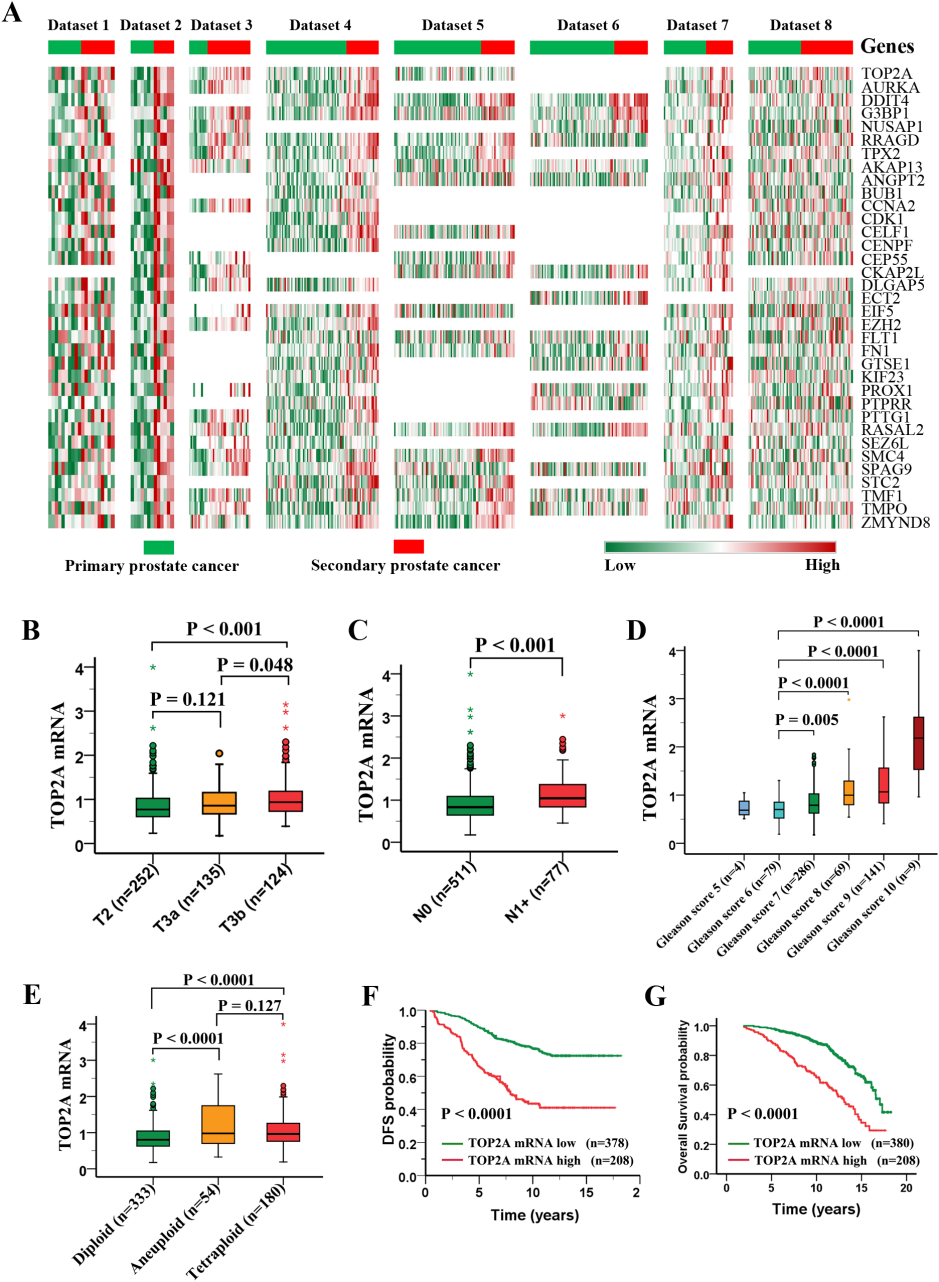
Profiling of upregulated genes in secondary prostate cancers and clinical significance of TOP2A expression in prostate cancer **(A)** Heat maps of 35 upregulated genes in more than four patient cohorts within dataset 1–8 (see Table 1). The most co-upregulated gene in 8 datasets is TOP2A. **(B-G)** Clinical significance of TOP2A expression in prostate cancer. The data were derived from the Nakagawa study (dataset 9). TOP2A overexpression is positively associated with the disease stages, lymph nodes metastasis, high Gleason score and chromosome abnormality. **(F and G)** Kaplan–Meier curve of disease-free survival (DFS) and overall survival (OS) in prostate cancer patients.

To find out candidate genes that are crucial for prostate cancer recurrence/metastasis, we analyzed 12 microarray datasets on prostate tumor studies (Table 1) and focused on the upregulated genes. The upregulated genes, duplication times and median fold changes in secondary prostate cancer relative to primary cancer are shown in Supplemental Excel file 1. Thirty-five genes were found upregulated in more than four patient cohorts and TOP2A ranked at the very top, which showed increased expression in 6 out of 8 datasets among these candidate genes (Figure 1A). Functional annotation of these upregulated genes using the DAVID revealed that they were mainly involved in mitosis, phosphorylation, cell cycle and cell division (Supplementary Figure 1A). To determine the impact of TOP2A expression on prostate cancer, we analyzed 4 larger cohorts of prostate cancer specimens with clinical annotations (see Table 1, datasets 9–12). TOP2A gene expression was correlated with advanced stages of the disease, higher Gleason scores, aneuploidy formation and poor survival in prostate cancer (Figure 1B-G; Supplementary Figure 1B-I). We then validated TOP2A protein expression in clinical prostatic tumors using immunohistochemistry. TOP2A was rarely detected in low-grade and post-castration prostate carcinomas but was expressed at high levels in high-grade or metastatic tumors (Supplementary Figure 2). As expected, we found that TOP2A expression was also positively correlated with the expression of cell proliferative marker Ki67 (see Supplementary Figure 2). Thus, TOP2A is a marker for secondary prostate cancer, and its overexpression correlates with high Gleason scores, metastasis and poor prognosis of prostate cancer.

**Table 1:** 12 prostate cancer datasets utilized to analyze gene signatures of secondary prostate cancer Dataset 1~8 were used for establishing an integrated dataset of secondary prostate model and 9~12 for the validation of the model.

Dataset	Author	Categorization	Source	Accession	Platform	Cases
**1**	Best	Androgen-independent prostate cancer	GEO	GSE2443 GDS1390	HG-U133A	20
**2**	Varambally	Metastatic prostate cancer	GEO	GSE3325 GDS1439	HG-U133_Plus_2	19
**3**	Hendriksen	Androgen-independent Xenograft	GEO	GSE4084 GDS2384	CMF/NKI produced Human 18K cDNA array	52
**4**	Yu & Chandra	Metastatic prostate cancer	GEO	GSE6919 GDS2545	HG_U95Av2	171
**5**	Yu & Chandra	Metastatic prostate cancer	GEO	GSE6919 GDS2546	HG_U95B	167
**6**	Yu & Chandra	Metastatic prostate cancer	GEO	GSE6919 GDS2547	HG_U95C	164
**7**	Tomlins	Metastatic/refractory prostate cancer	GEO	GSE6099 GDS3289	Chinnaiyan Human 20K Hs6	104
**8**	Sun	Recurrent prostate cancer	GEO	GSE25136 GDS4109	HG-U133A	79
**9**	Nakagawa	Systemic progression prostate cancer	Oncomine	GSE10645	Illumina DASL expression microarray	596
**10**	Grasso	Castrate Resistant Prostate Cancer	Oncomine	GSE35988	Agilent-014850 Whole Human Genome Microarray 4×44K	244
**11**	Setlur	Localized prostate	Oncomine	GSE8402	Human 6k Transcriptionally Informative Gene Panel for DASL	388
**12**	Taylor	Primary and metastatic prostate cancer	TCGA	GSE21032	Agilent-014693 Human Genome CGH Microarray	218

### Reduced proliferation but increased mesenchymal phenotype of DU145 cells upon TOP2A inhibition by shRNAs

**Figure 2:**
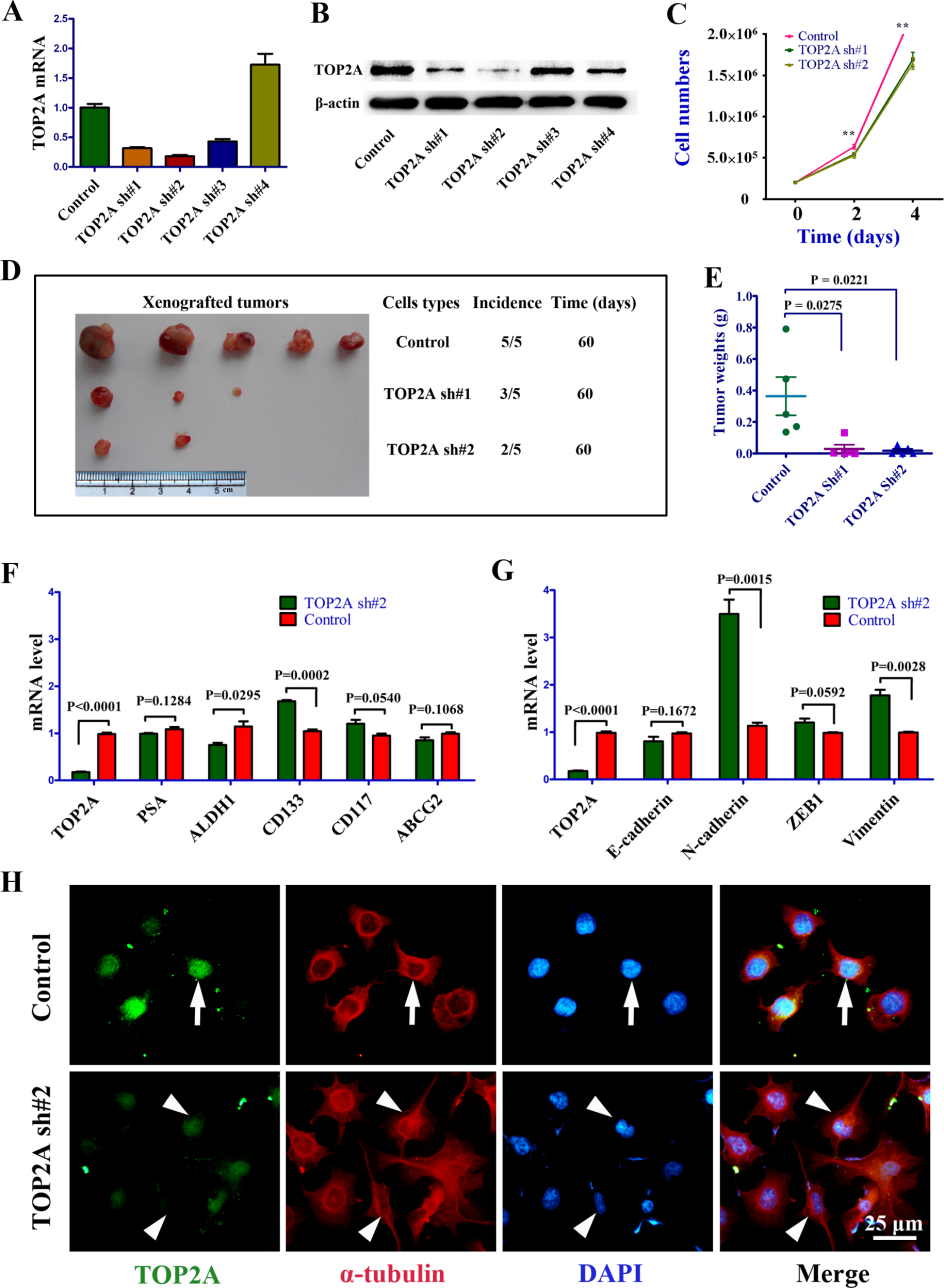
The phenotypes of DU145 cells following TOP2A inhibition by shRNAs **(A)** Silencing efficiency of four shRNAs targeting TOP2A as revealed by real-time PCR. **(B)** Western blot analysis to validate the TOP2A shRNA results of real-time PCR. **(C)** Cell growth curves of the control, TOP2A sh#1 and TOP2A sh#2 groups in DU145 cells. 2×10^5^ cells plated on 6-well plates and the cell numbers were counted by FACS every 2 days. Each group had three replications. **P < 0.01 by ANOVA followed by t-test. **(D and E)** Significantly decreased tumor incidence and growth by TOP2A shRNAs in a xenograft model. 1×10^4^ DU145 cells were implanted into nude mice in the three groups. **(F and G)** Analysis of prostate CSC and EMT associated gene expression by real-time PCR between the control and TOP2A sh#2 groups. **(H)** Morphological characteristics of EMT in DU145 cell after the inhibition of TOP2A by shRNA. When TOP2A were knocked down by TOP2A sh#2, fibrous morphology was observed in DU145 cells. Arrows show the cell with a normal shape in the control cultures. Arrowheads point to the cells displaying epithelial–mesenchymal transition (EMT) or a fibrous morphology in TOP2A sh#2 treated DU145 cells.

To gain further insight into the function of TOP2A in tumor cells, we knocked down TOP2A in DU145 cells. The two TOP2A shRNAs constructed dramatically reduced TOP2A expression at both mRNA and protein levels (Figure 2A and 2B). Cell proliferation and tumorigenicity were significantly reduced when TOP2A expression was inhibited by shRNA#1 or shRNA#2 in vitro and in vivo (Figure 2C-E). Intriguingly, several CSC markers including CD133 [11] and CD117 [25] were upregulated upon TOP2A knockdown (Figure 2F). Moreover, TOP2A knockdown induced molecular and morphological changes typical of EMT, including increased levels of N-cadherin and Vimentin, and appearance of the fibrous morphology in DU145 cells (Figure 2G-H; Supplementary Figure 3).

### Quiescence, smaller size, less apoptosis, and chemotherapy resistance of TOP2A^neg^ cells

**Figure 3:**
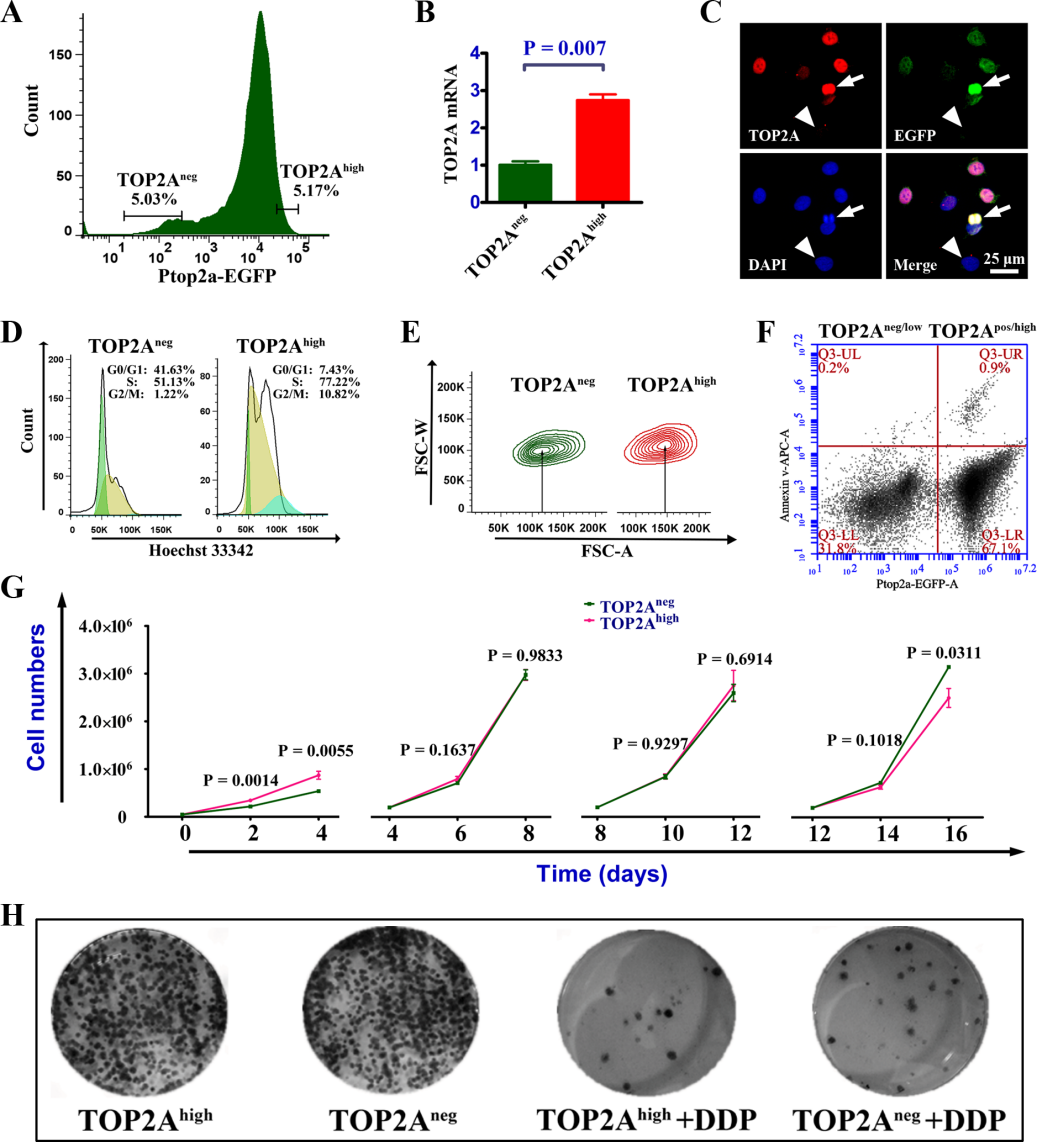
Cell cycle, proliferation and apoptosis analyses in TOP2A^neg^ and TOP2A^high^ cells **(A)** Flow cytometric determination of TOP2A^high^ and TOP2A^neg^ cells. Usually, the 5%-threshold was used for the cell sorting. **(B)** Real-time PCR shows that EGFP could faithfully reflect the expression of TOP2A in DU145 cells. **(C)** Immunofluorescent staining for TOP2A and EGFP to further validate the promoter report system. While arrows indicate the correlation between high expression of TOP2A and strong staining of EGFP, arrowheads show a control cell that is negative for both TOP2A and EGFP. **(D)** Cell cycle analysis between TOP2A^high^ and TOP2A^neg^ cells. **(E)** The comparison of cell volume between TOP2A^high^ and TOP2A^neg^ cells. **(F)** Apoptosis assay for TOP2A^neg/low^ and TOP2A^pos/high^ cells. **(G)** Proliferation assays following serial passages of TOP2A^high^ and TOP2A^neg^ cells. DU145 cells were subcultured every 2 days and the number of cells was adjusted to equal quantity for every 4 days. **(H)** Colony-forming assays of TOP2A^high^ and TOP2A^neg^ cells. There was little difference in clongenicity between TOP2A^high^ and TOP2A^neg^ cells whereas TOP2A^neg^ cells produced more clones than TOP2A^high^ cells after treatment with 2.5μmol DDP (cisplatin) for 3 days.

The above findings that knockdown of TOP2A using shRNA leads to upregulation of CSC markers and acquisition of mesenchymal phenotypes (stem cell–like phenotypes) implicate that TOP2A^neg^, rather than TOP2A^high^ cells behave as CSCs. We were then prompted to study whether TOP2A^neg^ cells possess other CSC features such as quiescence [26], smaller cell size [27], anti-apoptosis [28], resistance to chemotherapy [29], compared to TOP2A^high^ cells. Because TOP2A is not a cell surface marker, we generated a lentiviral reporter system in which EGFP and DsRed were driven separately by the TOP2A promoter and the CMV promoter. In this way, once the cells were infected, they would express DsRed. However, EGFP fluorescence intensities reflected the endogenous TOP2A gene expression (Supplementary Figure 4). TOP2A^high^ and TOP2A^neg^ cells were separated by FACS based on the intensity of green fluorescence (Figure 3A). Real-time PCR and immunofluorescent staining indicated that EGFP fluorescence intensities could faithfully represent the expression levels of TOP2A using this reporter system (Figure 3B and 3C). Cell cycle analysis revealed that a large portion of TOP2A^neg^ cells was in G0/G1 phase (quiescence phase), whereas more TOP2A^high^ cells were at the G2/M phase (10.82% compared with 1.22% in TOP2A^neg^ cells) (Figure 3D). Furthermore, FACS and annexin V analysis showed that TOP2A^neg^ cells had a smaller cell body size and less apoptosis than TOP2A^high^ cells (Figure 3E and 3F). In addition, TOP2A^high^ cells grew more quickly than TOP2A^neg^ cells in the first two passages based on cell number counting. However, at passage 3–6, there appeared no difference between the two populations. Importantly, by passage 7–8, TOP2A^neg^ cells grew more quickly than TOP2A^high^ cells (Figure 3G). These findings suggest that TOP2A^neg^ cells have a growth advantage over the TOP2A^high^ in the long run, which is in agreement with the features of CSCs, that is, lower apoptotic index and stronger proliferative potential [30]. Similar results were obtained when single cells were tracked using fluorescence microscope for 120 hours, which indicated that TOP2A^high^ cells could give rise to more daughter cells than TOP2A^neg^ cells in the short term (Supplementary Figure 5). However, colony-forming assay showed that there were no significantly differences at day 10 under normal culture conditions (Figure 3H, left). Interestingly, TOP2A^neg^ cells formed more colonies than TOP2A^high^ cells after cisplatin (DDP) administration for three days (Figure 3H, right). Collectively, these data indicate that TOP2A^neg^ cells display more CSC features than TOP2A^high^ cells.

### Higher capability of sphere formation, enrichment of side population and resistance to stress of TOP2A^neg^ cells

**Figure 4:**
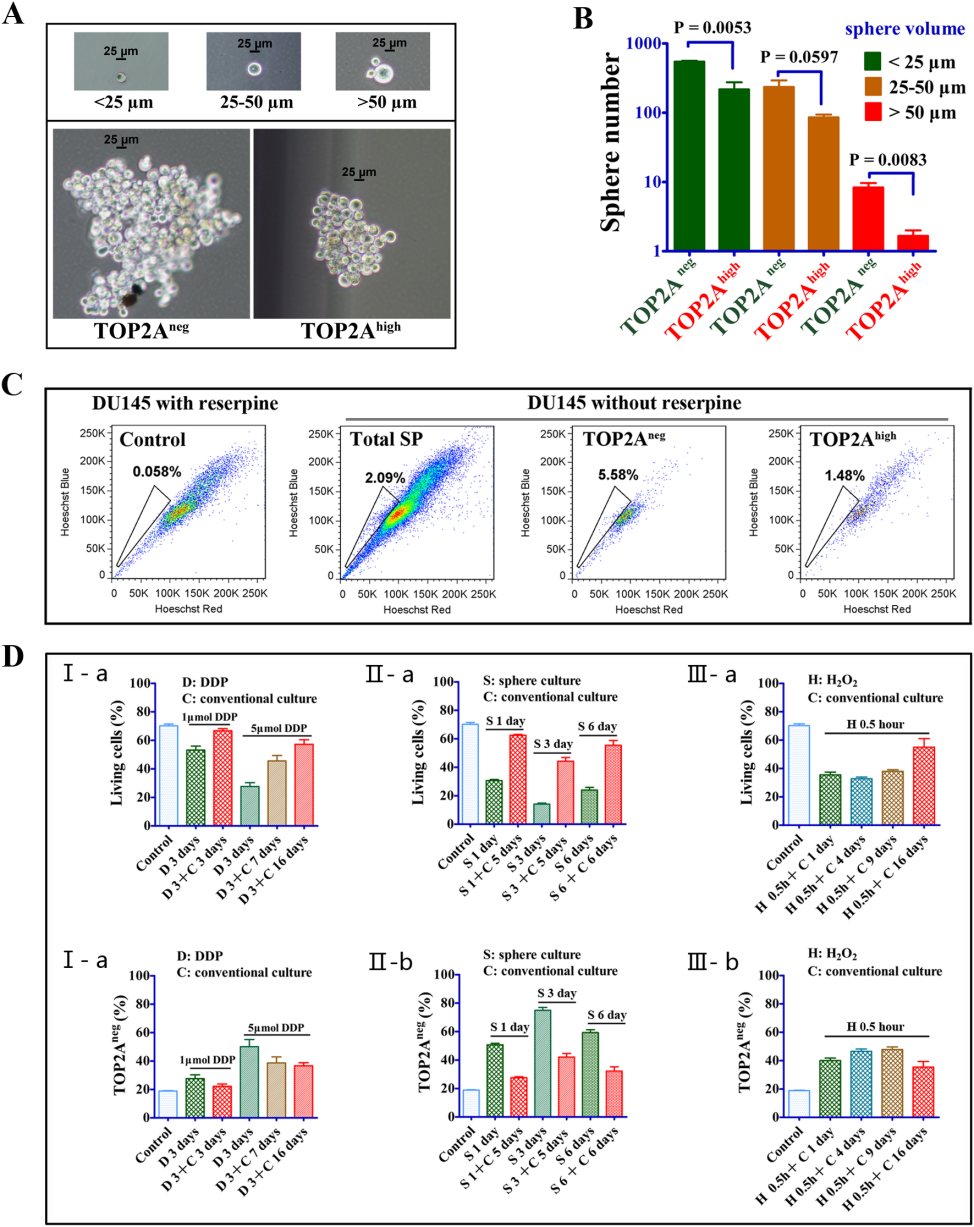
CSC features in TOP2A^neg^ cells **(A and B)** Sphere assays and statistical analysis of the sphere numbers and volumes between TOP2A^neg^ and TOP2A^high^ cells. **(C)** Side population (SP) enriched in TOP2A^neg^ cells. Reserpine is a chemical that prevents the expelling of Hoechst dye from the cells. DU145 cells were treated with 100 μmol/L reserpine before addition of Hoechst dye (left). The total SP cells, SP cells in TOP2A^neg^ and TOP2A^high^ cells were showed in the right. **(D)** The percentage change of living and TOP2A^neg^ cells in stressed environment. The proportion of living TOP2A^neg^ cells was analyzed after cells were treated by various stressed conditions and then returned to conventional culture. I, Cells were treated with 1 μmol/L or 5 μmol/L DDP for 3 days. II, Cells were cultured in the sphere medium (serum-free) for 1, 3 and 6 days. III, Cells were treated with 0.5mM H_2_O_2_ for 0.5 hours.

To provide additional evidence that TOP2A^neg^ cells represent CSCs, we performed sphere-formation, side population and resistance to stress assays with TOP2A^neg^ and TOP2A^high^ DU145 cells. As shown in Figure 4A and 4B, TOP2A^neg^ cells formed larger and more spheres than TOP2A^high^ cells. Furthermore, flow cytometry demonstrated that 5.58% of TOP2A^neg^ cells, but only 1.48% of TOP2A^high^ cells, possessed “side population” characteristics (Figure 4C), indicating an enrichment of CSCs in TOP2A^neg^ cell population. Moreover, because CSCs are considered to be resistant to stress [29, 31], we challenged TOP2A^neg^ cells against various types of stresses including DDP, serum-free and H_2_O_2_ treatments. Compared to the conventional cultures, the percentage of total live cells was decreased but the numbers of TOP2A^neg^ cells were significantly higher in the presence of stress environment. Additionally, the percentage of TOP2A^neg^ cells could be reversed following withdrawal of the stress (Figure 4D). Therefore, the percentage of the TOP2A^neg^ cells over the total population can be enriched under the stress, which further supports that TOP2A^neg^ cells have cancer stem cell characteristics.

### Dynamic changes and stronger tumorigenic potential of TOP2A^neg^ cells

**Figure 5:**
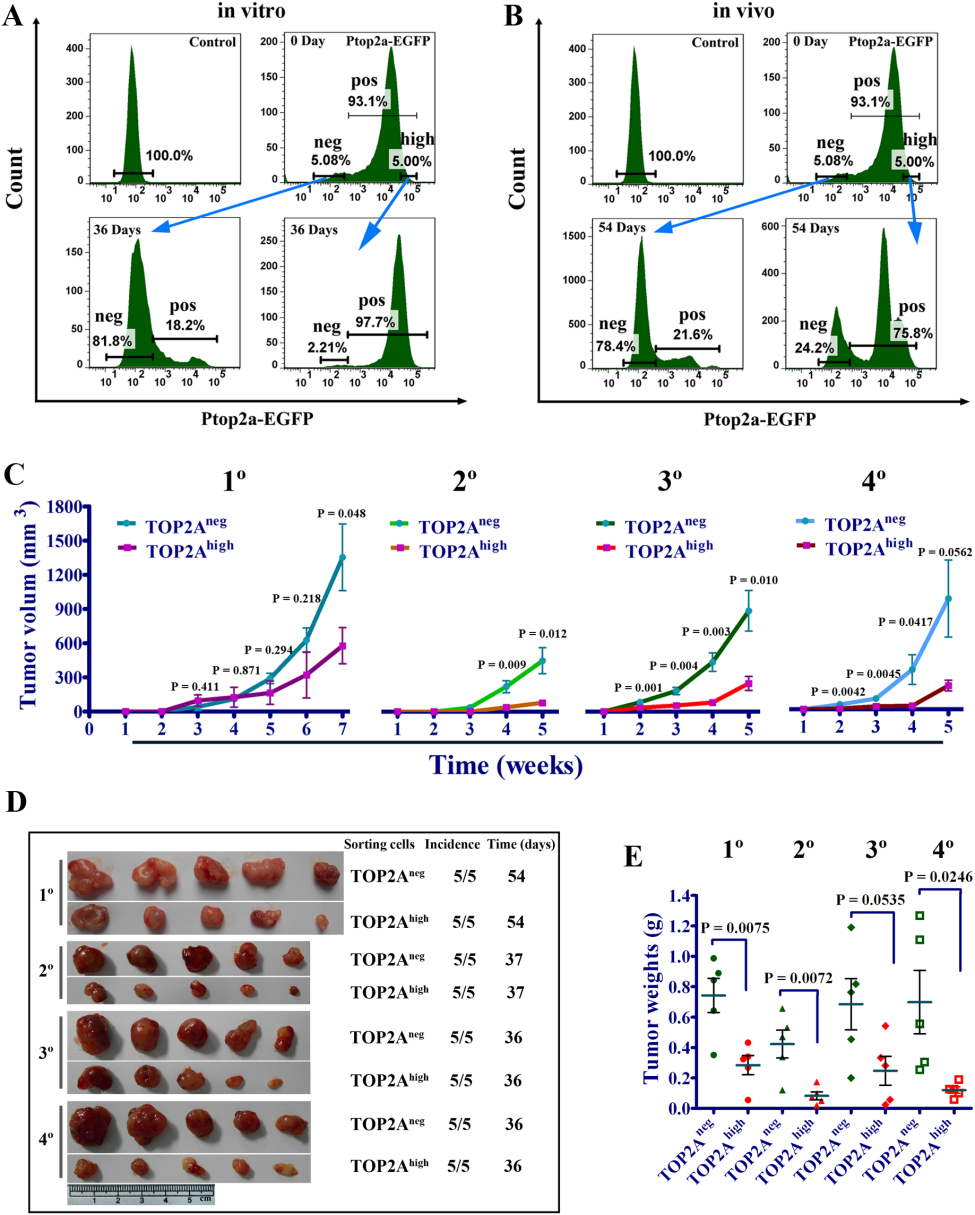
Dynamic changes and stronger tumorigenic potential of TOP2A^neg^ cells **(A)** The change in the proportion of TOP2A^neg^ and TOP2A^high^ cells as time proceeds in vitro. Ptop2a-EGFP cells were isolated by FACS according to EGFP levels as described previously using the maximum and minimum thresholds set at 5%. DU145 cells that were not infected with the lentiviral reporter were used as a control. **(B)** Dynamic changes of TOP2A^neg^ and TOP2A^pos^ cells in vivo. Xenograft tumors were finely minced and were digested with type IV collagenase (Sigma, 1 mg/ml) to obtain single-cell suspensions. Then the proportions of TOP2A^neg^ and TOP2A^pos^ cells were analyzed by FACS. **(C)** Tumor growth curves of serial xenograft tumors derived from 2×10^4^ TOP2A^neg^ and TOP2A^high^ cells. The 1° (first generation) tumors from TOP2A^high^ cells were digested to single cells and cultured for a week. Then TOP2A^neg^ and TOP2A^high^ cells sorted by FACS were implanted into nude mice for 2° (the second generation) tumor formation and so did the latter one for serial tumor transplantation. **(D and E)** Tumor formation and weight of tumors in the serial xenograft model using TOP2A^neg^ and TOP2A^high^ cells.

It was generally accepted by many researchers that CSC has unidirectional hierarchy and only tumorigenic cells could give rise to non-tumorigenic cells [30, 32, 33]. However, recent data suggest that CSCs manifest diverse plasticity [27, 34, 35]. To study the cell lineage relationship between TOP2A^neg^ and TOP2A^high^ cells, we tracked the two cell populations via real-time imaging in long-term cultures after cell sorting. Thirty-six days after TOP2A^neg^ and TOP2A^high^ cells were sorted by FACS and plated in the plates, TOP2A^neg^ cells gave rise to 18.2% TOP2A-positive cells. Intriguingly, TOP2A^high^ cells also generated 2.21% TOP2A^neg^ cells (Figure 5A). Consistently, 21.6% TOP2A-positive cells was observed by FACS analysis in vivo 54 days after TOP2A^neg^ cells were implanted, whereas 24.2% TOP2A^neg^ cells were also generated from TOP2A^high^ cells (Figure 5B). Thus the TOP2A^neg^ and TOP2A^high^ cells are reciprocally producible and there appears a plasticity of both CSCs and non-CSCs.

Since our studies have demonstrated TOP2A^high^ cells could form TOP2A^neg^ cells, we tried to explore whether TOP2A^neg^ cells derived from TOP2A^high^ cells could still keep cancer stem cell-like characteristics. Hence, we performed serial tumor transplantation experiments in nude mice with TOP2A^neg^ and TOP2A^high^ cells, in which both population were sorted from the same tumor generated by TOP2A^high^ cells. As shown in Figure 5C, although tumors derived from TOP2A^neg^ cells were smaller than those from TOP2A^high^ cells in the first four weeks in 1° (first generation) tumors, which was consistent with the proliferation assays in vitro (Figure 3G), both the volume and weight of the tumors derived from TOP2A^neg^ cells were markedly larger in the subsequent generations of tumors (Figure 5C-E). In addition, the size of the tumors derived from TOP2A^neg^ cells became bigger at 5 weeks along with serial transplantation, whereas the tumors generated from TOP2A^high^ cells were limited to a small size (Figure 5C). We also performed transplantation at limiting dilution using the first and fourth generation tumors, and found that TOP2A^neg^ cells had higher capacity to initiate tumor formation (Supplementary Figure 6). Therefore, limiting dilution and serial transplantation experiments reveal that TOP2A^neg^ cells represent CSCs, which are the driving force for sustained tumor growth.

### More aneuploidy formation in TOP2A^high^ cells and slower cell cycling in TOP2A^neg^ cells

**Figure 6:**
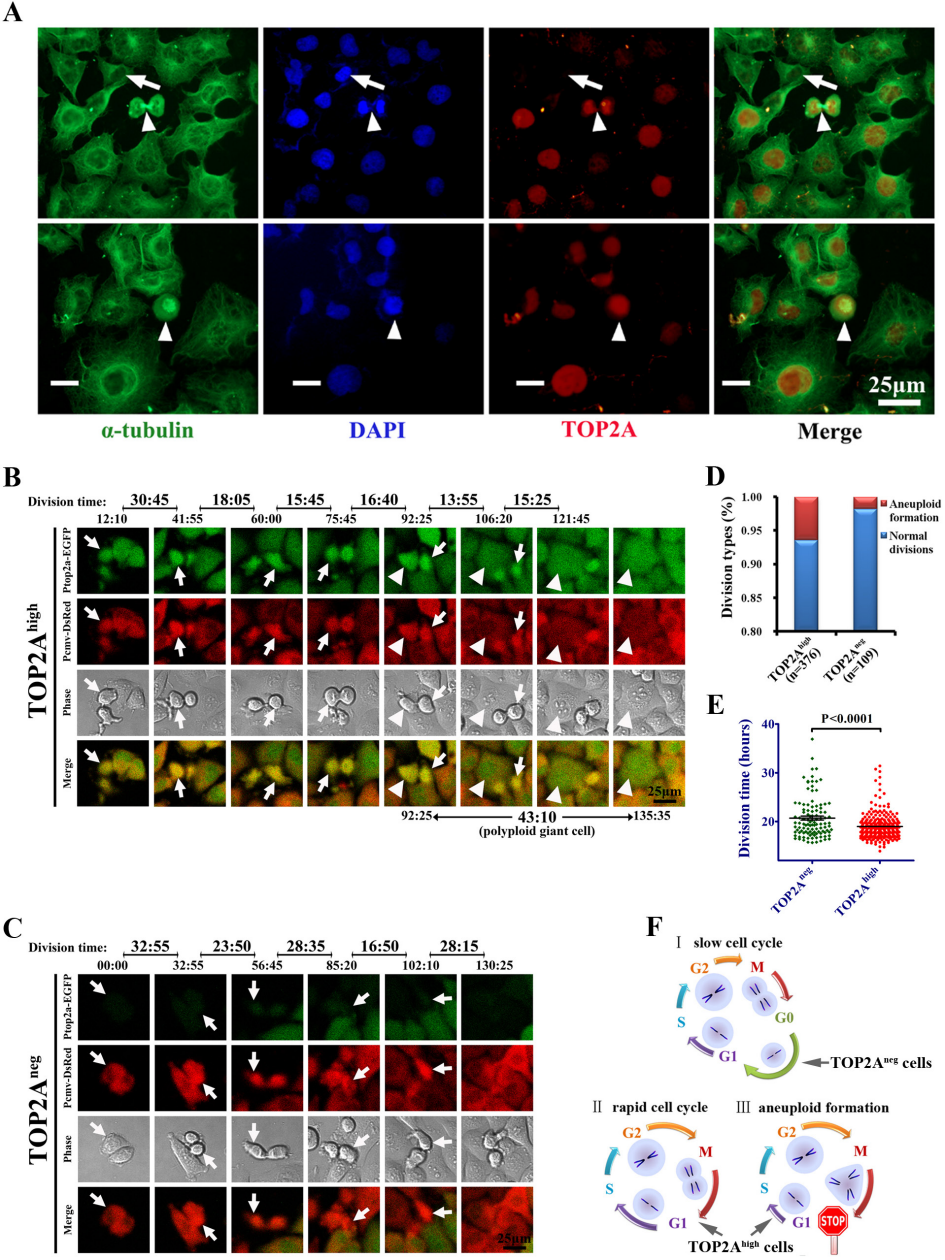
TOP2A expression and cell division analysis among DU145 cells **(A)** Expression of TOP2A in DU145 cells. Arrows indicate the negative staining in the cell with a small nucleus while arrowheads show high expression of TOP2A at M phase. Note that there is a polyploidy giant cell with strong staining of TOP2A at the bottom of the cell (white bar). **(B and C)** Time-lapse imaging system was used to monitor cell divisions in TOP2A^neg^ and TOP2A^high^ cells. Cell division events were tracked using EGFP, DsRed and DIC images and the detailed time course of divisions were recorded. Arrows indicate the parent cells dividing into two daughter cells while arrowheads show the formation of a polyploid giant cell with long cell cycle. **(D and E)** The proportion of aneuploid formation and division time in TOP2A^high^ and TOP2A^neg^ cells. **(F)** Schematic drawings to summarize the three types of cell divisions, including cells with slow cell cycle, cells undergoing a rapid division and cells displaying aneuploid formation.

Because maintenance of accurate cell division without aneuploidy is critical for the long-term stability and survival of CSCs, we next performed aneuploidy analysis and real-time imaging of cell division process to understand why TOP2A^neg^, but not TOP2A^high^ are CSCs. Immunofluorescence analyses for TOP2A and α-tubulin indicated that most cells had TOP2A expression and its expression was peaked at M phase, but there were a few cells that did not stain for TOP2A (Figure 6A). Interestingly, consistent with the results that aneuploidy formation is associated with higher level of TOP2A in clinical samples (Figure 1G), strong expression of TOP2A was also observed in the cell undergoing abnormal divisions (white bars in Figure 6A). Through analysis of the expression of TOP2A during various phases of the cell cycle, we found TOP2A was expressed at high levels at the metaphase of the cell cycle but low levels at interphase (Supplementary Figure 7A). These observations indicate that on one hand, TOP2A is a cell cycle-associated gene, which is in line with Figure 3D and previous reports [36, 37]. On the other hand, strong expression of TOP2A also occurs in the cells with aneuploidy formation including a giant nucleus, ternary fission and so on (Supplementary Figure 7B). These TOP2A^high^ cells with aneuploidy, might be more susceptible to apoptosis, and therefore are unlikely CSCs.

To further investigate the relationship between TOP2A^high^ cells and aneuploidy formation, we tracked cell divisions between TOP2A^high^ and TOP2A^neg^ cells at the single-cell level using time-lapse video microscopy. The results of cell tracking are shown in the Supplementary Movie. These real-time images revealed that TOP2A^high^ cells displayed a much shorter cell cycle than TOP2A^neg^ (Figure 6B and 6C). Notably, a TOP2A^high^ derived daughter cell formed a polyploidy giant cell and failed to undergo mitosis (white arrowhead in Figure 6B). The percentages of aneuploidy formation and doubling time of TOP2A^high^ and TOP2A^neg^ cells are shown in Figure 6D and 6E. These results indicated that TOP2A^high^ cells displayed a higher frequency of abnormal divisions while TOP2A^neg^ cells were slow-cycling cells. A schematic graph is shown to summarize the relationship between TOP2A expression and the patterns of cell divisions based on the above results (Figure 6F). In this model, TOP2A^neg^ cells have G0 phase and exhibit slow cell cycle while TOP2A^high^ cells are rapid proliferative cells but more susceptible to abnormal divisions. This model also gives a logical explanation why CSCs with a slow-cycling feature are not diluted by non-CSCs and do not disappear after serial passages in cell cultures.

## DISCUSSION

In this study, through systematic bioinformatic analyses, we have identified TOP2A as the most significantly upregulated gene in secondary prostate cancer. TOP2A encodes topoisomerase IIa (topoIIa), an enzyme that has been implicated in DNA topological structure and cell cycle progression [38, 39]. Previous reports indicate that TOP2A is a marker for proliferating cells in both normal and neoplastic tissues [40, 41]. Consistently, the present study showed that knocking down expression of TOP2A markedly suppresses cell proliferation in vitro and tumor growth in vivo. More importantly, we found that high expression of TOP2A correlates with advanced disease stages, tumor recurrence/metastasis and poor patient survival in human prostate cancer. Therefore, TOP2A is also an excellent prognostic marker for prostate cancer.

It has been well established that CSCs contribute to tumor relapse and metastasis, but it remains to be determined if the genes that play an essential role in tumor progression are true CSCs markers. Therefore, we explored the relationship of TOP2A expression and CSCs in prostate cancer. To our surprise, upon TOP2A inhibition by shRNA, we observed increased expression of CSC markers such as CD133 and CD117, as well as several mesenchymal genes including vimentin and N-cadherin. Furthermore, we confirmed that CSCs are enriched in TOP2A negative prostate cancer cells by directly sorting tumor cells based on their endogenous TOP2A expression followed by assessing sphere formation capability in vitro and sustained tumorigenesis in vivo. Although this seems counterintuitive considering recurrent tumors express higher levels of TOP2A, we propose that TOP2A only marks the proliferative state of tumor bulk while a small minority of TOP2A-negative, slow-cycling cells preserve more stemness capacity and tumor reinitiating capability. Our results also suggest that genes upregulated in relapsed or disseminated prostate cancer are not always true biomarkers of prostate CSCs. Similar data have been reported in other studies. For example, CD44^+^CD24^−/low^ population is tumorigenic with stem cell properties in breast cancer [15, 42], but CD24^+^ cells were dramatically enriched in distant metastases in breast cancer patients [43]. Furthermore, Wnt signaling pathway plays an important role in the maintenance of CSCs [44–46], but elevated expression of several Wnt target genes, including ASCL2 and LGR5, is actually associated with good prognosis in colorectal cancer [47].

Focusing on prostate CSCs enriched in TOP2A^neg^ cells, we further revealed several other intriguing properties of this subpopulation. First, TOP2A^high^ cells show more frequent abnormal cell divisions due to more aneuploidy formation. Second, cell division time of TOP2A^neg^ cells is significantly longer than TOP2A^high^ cells based on real-time imaging. Finally, TOP2A^neg^ CSCs are not static entities and exhibited considerable plasticity. Not only TOP2A^neg^ cells can give rise to TOP2A^high^ cells, some TOP2A^high^ cells also acquire stemness features and generate TOP2A^neg^ population both in vitro and in vivo. These data support a model of dynamic maintenance of cancer stemness [35, 48, 49], in which a TOP2A-negative, slow-cycling and self-renewing subpopulation of tumor cells constantly exists among the bulk of tumor cells. This model also explains why prostate cancer often progresses despite extensive treatments and imposes great challenges on future therapeutic strategies.

The results of our study reported here show that TOP2A is the phenotype of recurrence/metastasis in prostate cancer and a marker of rapid proliferation, whereas TOP2A^neg^ cells are CSCs and could regenerate TOP2A^high^ proliferative cells. As a result, therapies that target TOP2A^high^ cells (fast-proliferating cells) are urgent to reduce tumor burden and alleviate symptoms in patients with advanced prostate cancer. Our discovery is consistent with the theory proposed by Dr. Mikhail V. Blagosklonny, which advocates to target proliferating cancer cells and stemloids [50]. However, our study also demonstrated that low-cycling TOP2A^neg^ cells have features of CSCs in vitro and in vivo. Currently, most therapeutic regimens predominantly target the rapidly proliferating cancer cells [51]. Therefore, TOP2A^neg^ cells that are more resistant to conventional therapies could acquire additional mutations during cancer treatment, which may lead to cancer recurrence and/or metastasis. In conclusion, we believe that therapeutics targeting TOP2A negative cells, in combination with treatments to kill TOP2A positive cells, may provide a better method to eradicate primary prostate cancer.

## MATERIALS AND METHODS

### Upregulated genes in secondary prostate cancer

12 microarray datasets were accessed from GEO (Gene Expression Omnibus), TCGA (The Cancer Genome Atlas) and Oncomine to identify the profiles of secondary prostate cancer (see Table 1). Upregulated genes were obtained from the comparison between primary and secondary diseases. The integrated dataset of those genes was established by analyzing datasets 1–8, in which the top 1000 upregulated genes were selected from each dataset according to the median fold change. The most significantly differentially expressed genes in integrated datasets were entered into DAVID database (Database for Annotation, Visualization and Integrated Discovery, http://david.abcc.ncifcrf.gov/) for annotation. In addition, the reliability of upregulated genes in integrated datasets was subsequently validated in four independent patient cohorts (datasets 9–12).

### TOP2A shRNA used for knockdown experiment

Short hairpin RNA (shRNA) was used to knock down the expression of TOP2A. Four segments in TOP2A cDNA (NM_001067) were selected as shRNA targets. A separate shRNA for a nonspecific fragment was constructed as a negative control. The detailed position and sequence of the targeted region as well as the control shRNA are listed in Supplementary Table 1. The shRNAs were synthesized and subcloned into lentiviral vector (GV248) by Shanghai GeneChem Inc (Shanghai, China).

### Cell cultures and animals

DU145 prostate cancer cell line was purchased from ATCC (American Type Culture Collection), and cultured in DMEM supplemented with 10% heat-inactivated fetal bovine serum (FBS), 1% glutamine and 1% penicillin/streptomycin (Gibco BRL). BALB/c nude mice were obtained from SLACCAS (Shanghai Laboratory Animal Center), bred in our own animal facility and maintained under standard conditions according to the institutional guidelines of Ren Ji Hospital animal care and ethics review committee.

### Quantitative real-time PCR

RNA samples were reverse transcribed using PrimeScript II 1st Strand cDNA Synthesis Kit (Takara Bio Inc., Japan). Real-time PCR was performed in triplicates, and each 20 μL reaction contained 2×SYBR Green Master Mix (Toyobo, Japan), 1 μL forward and reverse primers, and 2 μL of cDNA template. Samples were amplified using the StepOne Plus Real Time PCR System (Applied Biosystems, Norwalk, CT, USA). The amplification conditions were 95°C for 2 min followed by 40 cycles of 95°C for 15 s and 60°C for 1 min. Gene expression was presented using the comparative CT method (2^−ΔΔCT^)[52]. The sequences of forward and reverse primers used for real-time PCR were shown in Supplementary Table 2.

### Construction of promoter reporter system

TOP2A promoter region was amplified from genomic DNA of DU145 cells by PCR with specific primer sets (forward 5′-TACTGTCAGCCCACTGTTTACC-3′; reverse 5′-TACTGTCAGCCCACTGTTTACC-3′). Amplified fragments were constructed into the lentiviral vector (Addgene plasmid 24526), in which the UBC promoter was replaced by the TOP2A promoter. Then cells infected with virus were sorted to isolate TOP2A^high^ and TOP2A^neg^ cells by FACS (FACS; Becton Dickinson) according to the fluorescence intensity of enhanced green fluorescent protein (EGFP). The TOP2A expression of TOP2A^high^ and TOP2A^neg^ cells was analyzed by real-time PCR and immunofluorescence to confirm if EGFP faithfully reflected the expression of TOP2A in the lentiviral reporter system.

### Cell cycle and size determination

DU145 cells infected with the reporter lentivirus were cultured in T75 flasks and harvested at 75% confluence. Cells were stained with Hoechst 33342 dye (Molecular Probes-Invitrogen) at a concentration of 2μg/mL for 15 minutes. The fraction of cells in G0/G1, S, or G2/M phase were determined by FACS between TOP2A^high^ and TOP2A^neg^ cells. In addition, forward scatter (FSC) and side scatter (SSC) were used to measure the size of these cells.

### Cell proliferation and apopotosis assays

Suspended cells were plated in 6-well plates and the numbers of surviving cells were counted every 2 days by FACS. For serial proliferation assays, cells were subcultured every 2 days and the number of cells was adjusted to ensure the equal quantity every 4 days. To assess cellular apoptosis in vitro, 1×10^6^ suspended cells were stained with Annexin-APC (KeyGen Biotech Co., Nanjing, China) and the percentage of apoptotic cells was measured by flow cytometry.

### Colony-forming assay

TOP2A^high^ and TOP2A^neg^ cells were sorted and suspended into 6-well plates at low density (1500 cell/well) to determine their clone abilities. All samples were plated in triplicates and cultured for 10 days. Then cells were fixed in 4% PFA and stained by 0.1% crystal violet. The colony-forming efficiency was evaluated by the numbers of colonies.

### Sphere-forming assay

Two thousand sorted cells were cultured in serum-free medium (SFM) in low attachment 6-well plates (Corning Costar). The medium was DMEM/F2 supplemented with 20ng/mL human epidermal growth factor (EGF; Sigma-Aldrich) and 20ng/mL human basic fibroblast growth factor (bFGF; Sigma-Aldrich). Cells were cultured for 7~10 days and the number and volume of spheres were counted under an optical microscope (100X).

### Side population analysis

This experiment was based on Goodell et al with modifications [53]. Briefly, DU145 cells (1×10^6^ cells/mL) were incubated in DMEM containing 10% FBS and added Hoechst 33342 (5μg /mL final concentration) for 90 minutes at 37°C. In the control group, 100 μmol/L reserpine were added to prevent the expelling of Hoechst from the cells. The Hoechst dye was excited with the UV laser at 355 nm and its fluorescence was dual-wavelength analyzed (blue, 402–446 nm; red, 650–670 nm).

### Immunohistochemistry

Immunohistochemistry (IHC) of formalin-fixed paraffin-embedded human prostate cancer tissues was carried out to further validate interesting genes in the secondary diseases. Those samples were collected from the prostate cancer patients with the primary tumors, following castration, displaying lymph node metastasis and systemic metastastasis. All of the cancer tissues were collected from Renji Hospital, shanghai and these samples for IHC were approved by the institutional ethics review committee of the Renji Hospital, Shanghai Jiao Tong University School of Medicine.

### Immunofluorescence

DU145 cells cultured on sterile glass cover slips were fixed with 4% formaldehyde for 10 min. Then cells were incubated in permeabilization buffer (0.3% Triton X100, in PBS) for 10 minutes. Ten percent donkey serum was added to suppress nonspecific antibody binding at room temperature for 30 minutes and primary antibodies were incubated overnight at 4°C. Fluorochrome-conjugated secondary antibodies were added after wash in a dark chamber at room temperature. The slides were subsequently washed with PBS and mounted using mounting solution containing DAPI. Finally, slides were visualized with a NIKON80i fluorescent microscope. The information of antibodies applied in experiments is shown in Supplementary Table 3.

### Western blot

For immunoblotting, equal amounts of proteins (10–50μg) were subjected to SDS/PAGE on 10%–12% SDS-PAGE and electrophoretically transferred onto polyvinylidene difluoride (PVDF) membrane (Millipore). The blots were blocked for 1 hour with TBST (50 mM Tris-HCl 150 mM; NaCl, 0.3% Triton X-100, pH, 7.6) containing 5% nonfat dry milk at room temperature, and then it was incubated with primary antibody overnight at 4°C. The primary antibodies were detected using secondary antibody (Goat anti-Rabbit or Mouse HRP conjugate), which were diluted in TBST containing 5% nonfat dry milk. The immunocomplexes were visualized with Immobilon Western Chemiluminescent HRP Substrate (Millipore) and photographed with Chemi-Doc™ XRS System (BIO-RAD, USA). The detail information of antibodies applied in Western blot is also shown in Supplementary Table 3.

### Xenograft model and tumorigenicity

DU145 cells were suspended in basic medium and mixed with Matrigel (BD Biosciences) at a 1:1 ratio and total volume of 150 μL mixture was subcutaneously injected into the flanks of BALB/c nude mice. Tumor growth was monitored every 1 week after implantation and the volume of tumor was calculated by the formula 0.5×length×width^2^. Animals were sacrificed and the tumors were weighed before the largest diameter of tumors reached at 2.0 cm to prevent tumor necrosis. For serial tumor formation, the first generation tumors were finely minced and were digested with type IV collagenase (Sigma, 1 mg/mL) to obtain single-cell suspensions. It was cultured for one week and then TOP2A^neg^ and TOP2A^high^ cells were sorted again to implant into nude mice.

### Live cell imaging

Time-lapse videomicroscopy was used to observe cell divisions of infected cells. Cells were plated on glass-bottom dishes at low confluence in the incubator of Nikon Biostation Timelapse system. The incubator was maintained at 37°C and with >95% humidity. Differential interference contrast (DIC), EGFP and Discosoma red fluorescent protein (DsRed) images were captured continuously with a 20X objective lens at a 5 min interval for 3–7 days. Then the images were analyzed by the Nikon NIS-Elements software.

### Statistical analyses

Statistical analysis was carried out using SPSS version 16.0 (SPSS, Inc., Chicago, IL, USA). Statistical differences were analyzed by the Student's t test. All tests of statistical significance were two-sided and the 0.05 level of significance was used for all data analyses.

## SUPPLEMENTARY FIGURES AND TABLES




