# Supplementary material for: TOP2Ahigh is the phenotype of recurrence and metastasis whereas TOP2Aneg cells represent cancer stem cells in prostate cancer
Source: Oncotarget. 2014 Sep 8;5(19):9498–513. doi: 10.18632/oncotarget.2411 (PMC4253449; doi:10.18632/oncotarget.2411)
Supplement: Supplementary file 1 [file oncotarget-05-9498-s001.pdf]

SUPPLEMENTARY FIGURES AND TABLES

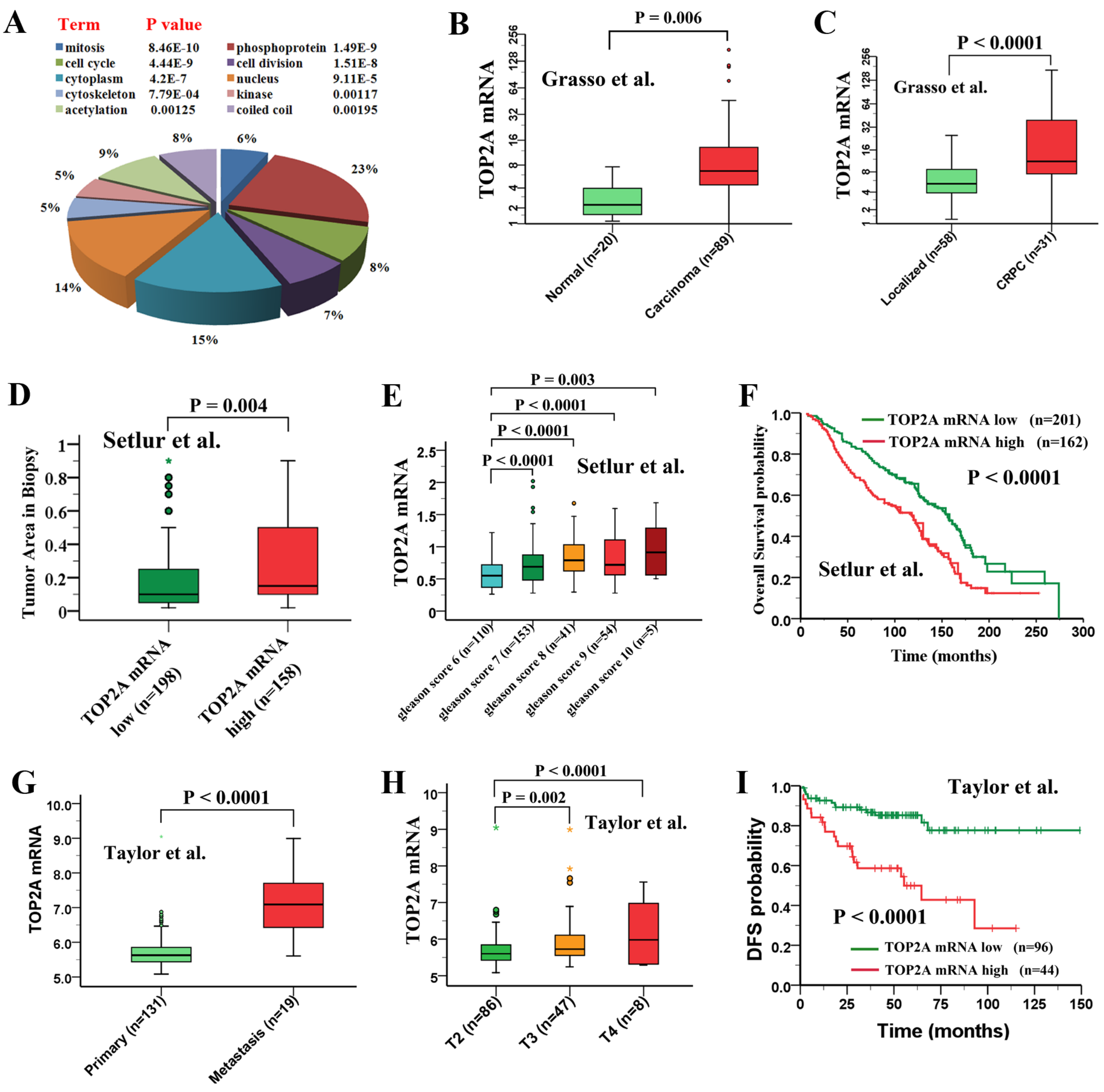

Supplementary Figure S1: The features of secondary prostate cancer and TOP2A expression in three additional independent studies. **(A)** The features of secondary prostate cancer annotated by DAVID. Thirthy-five upregulated genes in secondary prostate cancer were analyzed for functional annotation by DAVID. **(B-I)** The relationship of TOP2A expression and clinical characteristics among prostate cancer patients were confirmed by Grasso, Setlur and Taylor studies (datasets 10–12).

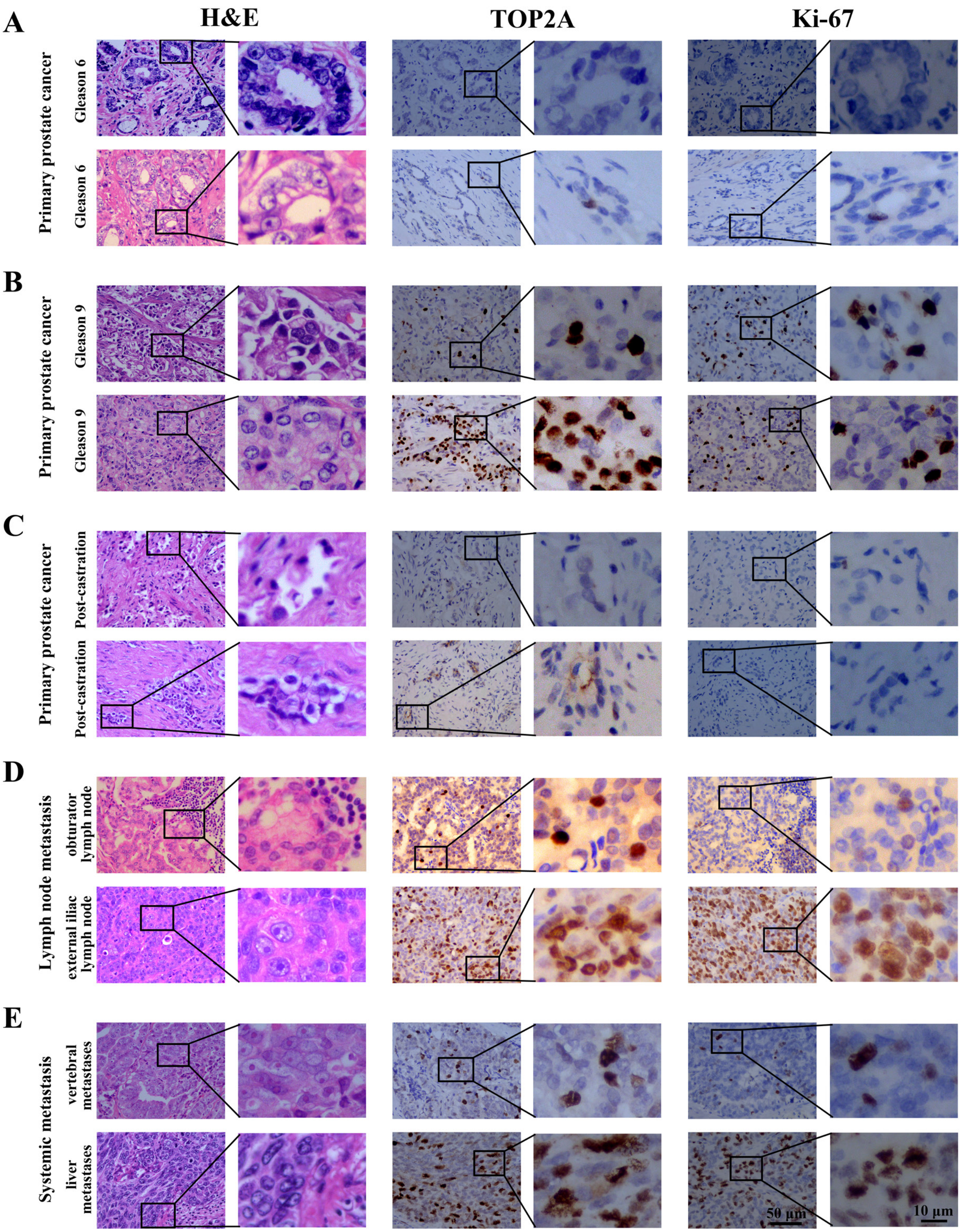

Supplementary Figure S2: Representative images from sections stained with H&E (hematoxylin and eosin) and immunohistochemical analysis for TOP2A and Ki-67 in clinical prostate tumor samples. **(A and C)** No or few cells are stained for TOP2A and Ki-67 in low grade (Gleason 6) and castration prostate carcinoma. **(B, D and E)** Much more cells are stained for TOP2A and Ki-67 in high grade (Gleason 9), lymph node metastasis and systemic metastasis prostate cancer.

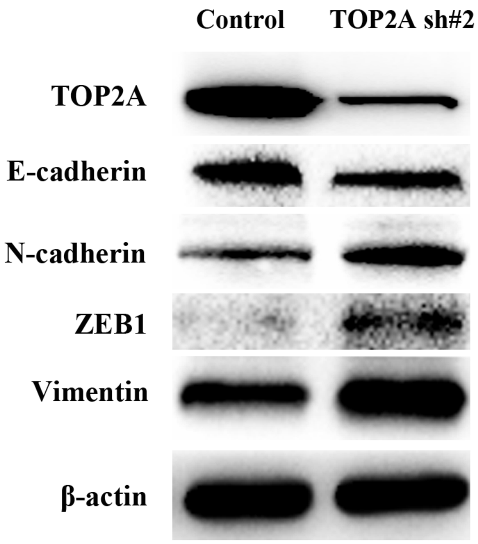

Supplementary Figure S3: EMT markers were detected by Western blot analysis between control and TOP2A sh#2 groups.

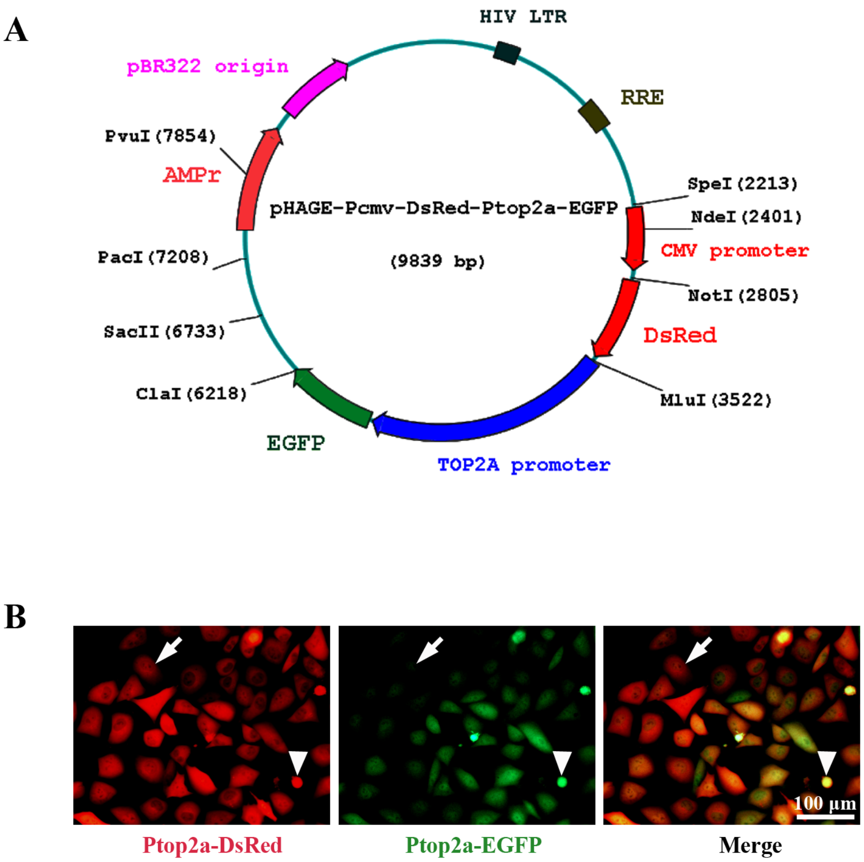

Supplementary Figure S4: The construction of the lentiviral reporter system. **(A)** The map of lentiviral vector utilized to isolate and to track TOP2A^{high} and TOP2A^{neg} cells. **(B)** Photographs for TOP2A^{high} and TOP2A^{neg} cells under a fluorescence microscope. DU145 cells infected with the virus were sorted firstly by FACS according to the constitutively expressed DsRed, which was driven by the CMV promoter and was used as an indicator for successful viral infection and integration. Then cells were plated in 6-well plates at low density and the expression of DsRed and EGFP were observed to validate the promoter reporter system. Arrows demonstrate that there is no expression of EGFP in the cell while DsRed have been observed. Arrowheads indicate a dividing cell with the expression of both EGFP and DsRed.

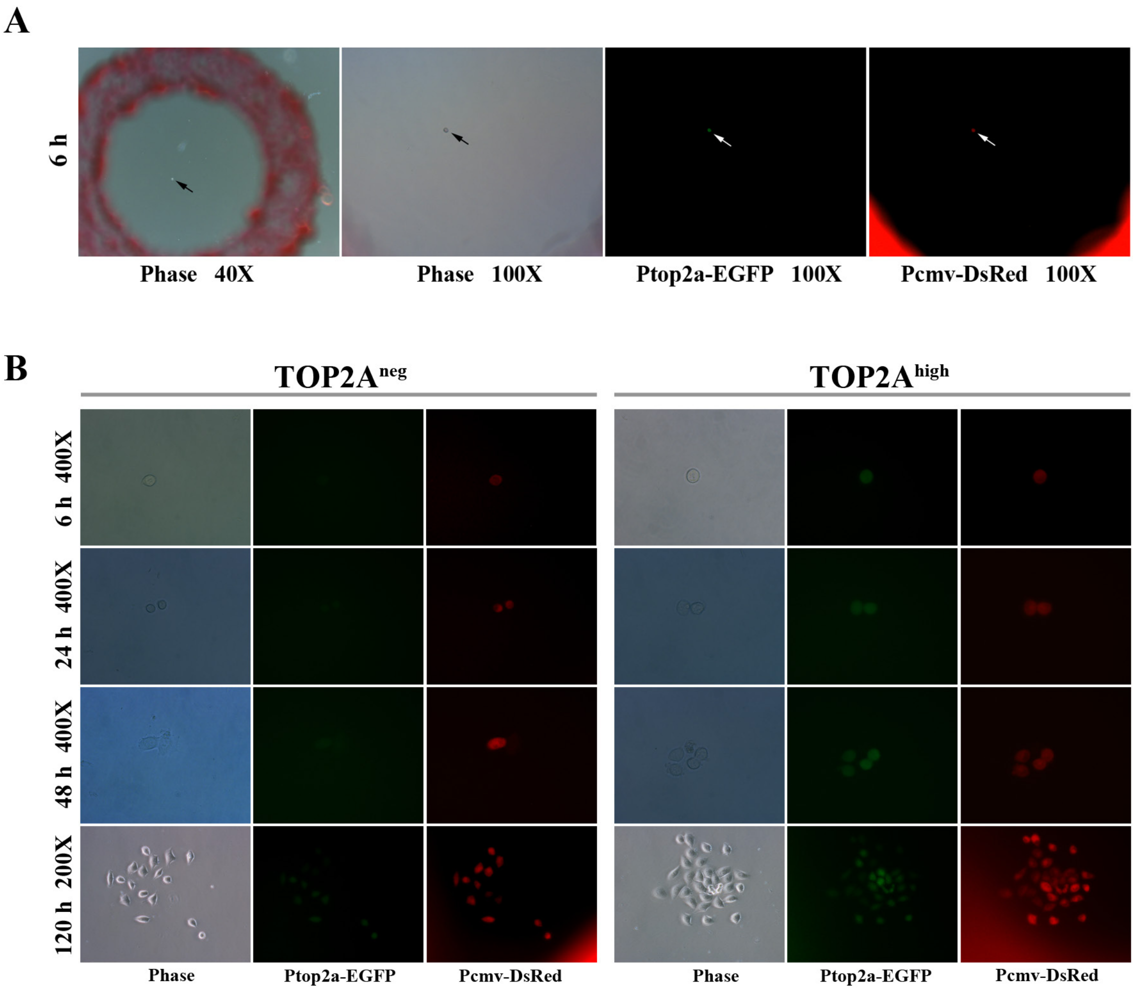

Supplementary Figure S5: Single TOP2A^{neg} and TOP2A^{high} cells were tracked under a fluorescent microscope. **(A)** Single cell was stamped and observed when sorted cells were plated into T-75 plastic culture flask for 6 hours. **(B)** Images were taken at different time points to track the proliferation of TOP2A^{neg} and TOP2A^{high} cells.

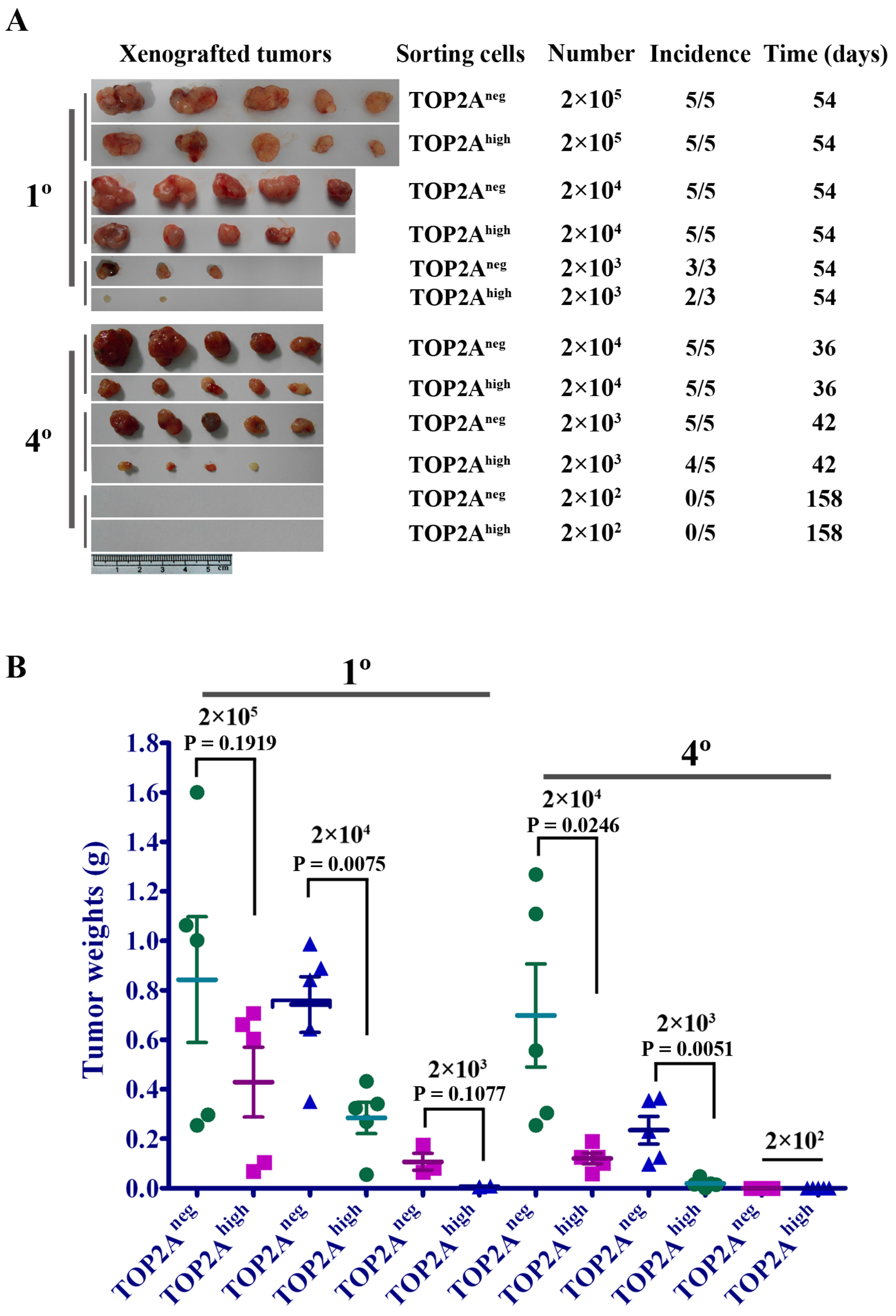

Supplementary Figure S6: Limiting dilution transplantation of TOP2A^{neg} and TOP2A^{high} cells. **(A)** Photograph of first and fourth generation tumors that were developed in nude mice. TOP2A^{neg} and TOP2A^{high} cells were isolated and 2×10^2 ~ 2×10^5 cells were implanted subcutaneously in nude mice. **(B)** The statistical analysis of tumor weights. Xenografted tumors derived from TOP2A^{neg} and TOP2A^{high} cells were measured and their weights were analysed by non-paired Student's test.

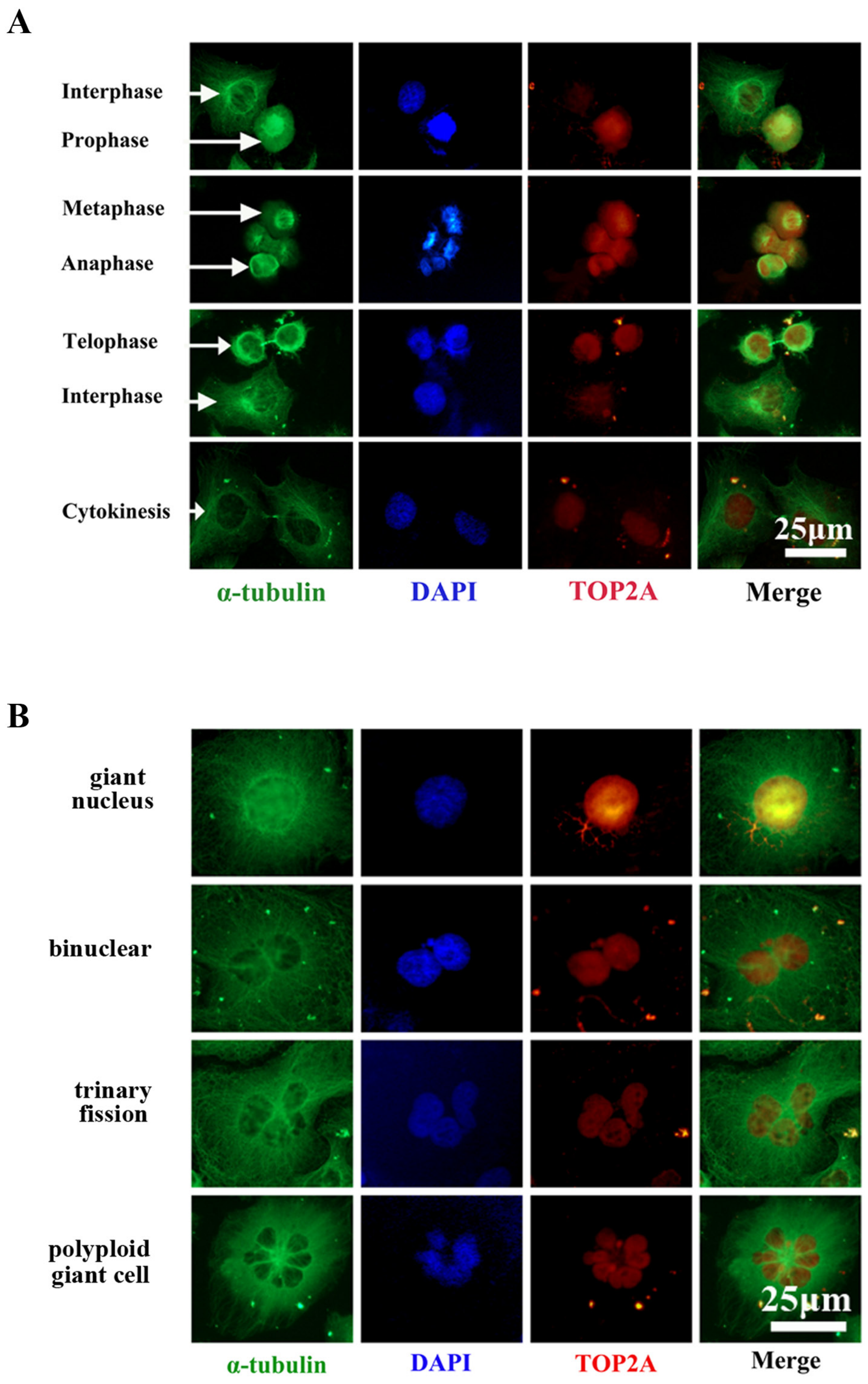

Supplementary Figure S7: Immunofluorescent staining for TOP2A and α-tubulin in DU145 cells. **(A)** Dynamic expression of TOP2A during a cell cycle. **(B)** High expression of TOP2A in DU145 cells that undergoes abnormal divisions. The abnormal cell divisions are defined based on the feature of a polyploidy giant nucleus, binuclear, ternary fission and polyploidy giant cell.

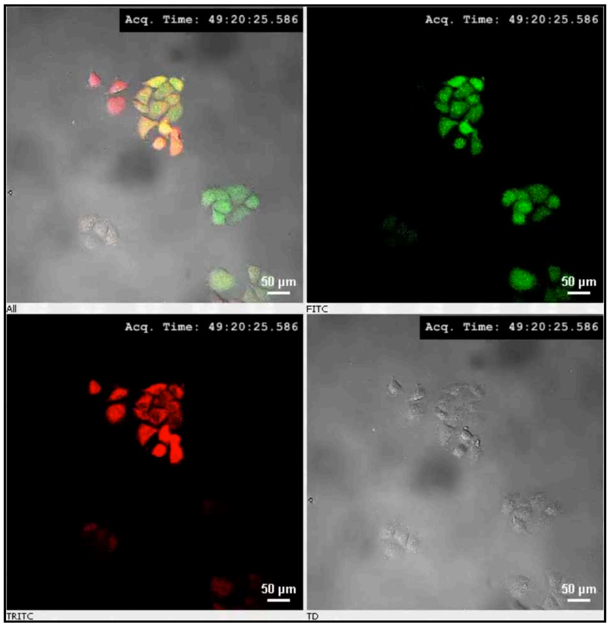

Supplementary Movie: Time-lapse imaging of DU145 cells to track their divisions from TOP2Aneg and TOP2Ahigh cells. Details of the protocol have been described in the parts related to construction of promoter reporter system and live cell imaging (See MATERIALS AND METHODS). Time course in the upper right corner is displayed as h:m:s.ms.

Supplementary Table S1. Candidate TOP2A Short hairpin RNAs (shRNAs) used in knockdown experiments. This table shows the detail position and sequence of the targeted region.

ID	Position in targeted region	Sequence of the targeted region
**TOP2A-sh#1**	4897–4915	GCCCAAGTGTTCTTTAGCTTT
**TOP2A- sh#2**	821–839	GCCTGATTTGTCTAAGTTTAA
**TOP2A- sh#3**	2868–2886	GCTCCAAATCAATATGTGATT
**TOP2A- sh#4**	2754–2772	CCCAACTTTGATGTGCGTGAA
**TOP2A- sh#neg / Control**	Nonspecific control	TTCTCCGAACGTGTCACGT

Supplementary Table S2. Sequences of the primers designed to analyze prostate CSCs and EMT markers by real-time PCR.

Gene	Sequence (5′-3′)
TOP2a-F	ACAAGACATCAAAGTGAAGTAAAGCC
TOP2a-R	GCAGACTCAAAACACAGACAAAGC
β-actin-F	GGTGACAGCAGTCGGTTGGAG
β-actin-R	GAGAAGTGGGGTGGCTTTTAGG
PSA-F	AGGCACAACGCACCAGACAC
PSA-R	CCTCCCTCCTTGGCTCACAG
ALDH1-F	CCGTGGCGTACTATGGATGC
ALDH1-R	GCAGCAGACGATCTCTTTCGAT
CD133-F	AGTCGGAAACTGGCAGATAGC
CD133-R	GGTAGTGTTGTACTGGGCCAAT
CD117-F	ACTTGAGGTTTATTCCTGACCCC
CD117-R	GCAGACAGAGCCGATGGTAG
ABCG2-F	TGAGCCTACAACTGGCTTAGA
ABCG2-R	CCCTGCTTAGACATCCTTTTCAG
E-Cadherin-F	CGAGAGCTACACGTTCACGG
E-Cadherin-R	GGGTGTCGAGGGAAAAATAGG
ZEB1-F	GATGATGAATGCGAGTCAGATGC
ZEB1-R	ACAGCAGTGTCTTGTTGTTGT
N-Cadherin-F	TCAGGCGTCTGTAGAGGCTT
N-Cadherin-R	ATGCACATCCTTCGATAAGACTG
Vimentin-F	AGTCCACTGAGTACCGGAGAC
Vimentin-R	CATTTCACGCATCTGGCGTTC

Supplementary Table S3. Antibodies used for immunofluorescence and western blot.

Primary antibodies

Antibody (species)	Catalog #	Supplier	Unit	Application	Dilution
TOP2A (goat)	sc-5346	Santa Cruz Biotechnology	200µg/ml	Immunofluorescence	1:100
α-tubulin (mouse)	66031-1-Ig	ProteinTech Group	167µg/150µl	Immunofluorescence	1:400
EGFP (rabbit)	ab290	abcam	5mg/ml	Immunofluorescence	1:200
ki-67 (rabbit)	AB54094	Sangon Biotech	2.76mg/ml	Immunofluorescence	1:200
TOP2A (rabblit)	20233-1-AP	ProteinTech Group	28 µg/150 µl	Western blot	1:300
E-Cadherin(mouse)	610181	BD Biosciences	250 µg/ml	Western blot	1:500
N-Cadherin(rabblit)	610920	BD Biosciences	250 µg/ml	Western blot	1:500
Vimentin(rabbit)	2701-1	Epitomics	100µl	Western blot	1:1000
ZEB1 (rabbit)	3396S	CST	100µl	Western blot	1:500

Secondary antibodies

Name	Catalog #	Supplier	Unit	Application	Dilution
Alexa Fluor® 488 donkey anti–goat	A-11055	Life Technologies	2 mg/ml	Immunofluorescence	1:500
Alexa Fluor® 594 donkey anti–goat	A-11058	Life Technologies	2 mg/ml	Immunofluorescence	1:500
Alexa Fluor® 488 donkey anti–mouse	A-21202	Life Technologies	2 mg/ml	Immunofluorescence	1:500
Alexa Fluor® 546 donkey anti–mouse	A-10036	Life Technologies	2 mg/ml	Immunofluorescence	1:500
Alexa Fluor® 594 donkey anti–rabbit	A-21207	Life Technologies	2 mg/ml	Immunofluorescence	1:500
Goat Anti-Mouse IgG (H+L) HRP	AP124P	Millipore	2.0ml	Western blot	1:7000
Goat Anti-Rabbit IgG (H+L) HRP	AP123P	Millipore	2.0ml	Western blot	1:7000
